# Detection of Melamine in Feed Using Liquid-Liquid Extraction Treatment Combined with Surface-Enhanced Raman Scattering Spectroscopy

**DOI:** 10.1371/journal.pone.0107770

**Published:** 2014-09-22

**Authors:** Jie Cheng, Shi Wang, Xiao-Ou Su

**Affiliations:** Institute of Quality Standards and Testing Technologies for Agro-products, Chinese Academy of Agricultural Sciences, Beijing, China; University of Georgia, United States of America

## Abstract

A rapid, selective, and sensitive method to determine the melamine content in animal feeds was developed using surface-enhanced Raman scattering spectroscopy on aggregated 55 nm Au nanoparticles with liquid–liquid extraction sample preparation. Butyl alcohol was used as the initial extraction solvent, and liquid–liquid extraction was performed twice using HCl (pH 3–4) and 6∶1 (v/v) *n*-butyl alcohol/ethyl acetate. The intensity of the matrix-based peak at 731 cm^−1^ was set at 100 as a basis for the feeds, and the peak at 707 cm^−1^ was the characteristic peak of melamine used in the calculations. Sufficient linearity was obtained in the range 2–10 µg·g^−1^ (*R* ^2^ = 0.991). Limits of detection and quantification in the feeds were 0.5 and 2 µg·g^−1^, respectively. The recovery rates were 82.5–90.2% with coefficients of variation below 4.02%. This new protocol could be easily developed for the routine monitoring of on-site feed quality and market surveillance.

## Introduction

Melamine (1,3,5-triazine-2,4,6-triamine, C_3_H_6_N_6_) is mainly used in the production of melamine-formaldehyde resins which are utilized as coatings, glues, laminates, and heat-tolerant polymers [1]–[4]. However, because of its high nitrogen content (66.7% by mass), melamine is illegally added to animal feeds to increase their apparent protein contents, because the protein levels are estimated by the conventional Kjeldahl or Dumas methods. A reported outbreak of pet deaths in the United States was traced to melamine-induced kidney failure caused by adulterated feed [5]. In the U.S., swine and livestock feeds have also been found to be contaminated to some extent [6]. Though various toxicology studies have found that melamine toxicity in mammals is very low [7], the risk assessment for melamine is complicated by the fact that it can exert differential toxicity in various animal species [8]. When melamine and cyanuric acid (a constituent of urine) combine, they form white melamine cyanurate crystals that instantly precipitate in the kidneys, thus causing renal failure. The U.S. Food and Drug Administration, the European Union, and agencies in other countries have established maximum residual limits of melamine in animal feed. Generally, a threshold of 2.5 µg·g^−1^ is accepted by most countries.

Although melamine has been found only in the wheat gluten, rice protein concentrate, and corn gluten used in animal feed, it cannot be assumed that melamine has not been added to other protein sources intended to be used as feed [9]. In this regard, the development of effective methods to monitor melamine in animal feed has become necessary and urgent, and therefore, it is worth investigating both the current and alternative techniques used to detect and determine the concentration of melamine in feed. Methods such as gas chromatography/mass spectrometry (GC/MS) [10] and liquid chromatography–tandem mass spectrometry [11] are commonly used in the analysis of melamine in feeds or feedstuffs. However, these methods require complicated sample-preparation processes, clean-up steps, and high-cost instruments, which make the tests difficult to implement widely. Various fast techniques have been developed for melamine detection, namely, enzyme-linked immunosorbent assay [12] and near-infrared reflectance spectroscopy [13]. However, these also require complicated instrumentation and highly trained technical staff, which limit their application. With the developments in nanotechnology, studies based on surface-enhanced Raman spectroscopy (SERS) for melamine have been conducted [14]. Though SERS exhibits high selectivity for melamine, improving the reproducibility of SERS intensity measurements and developing relatively simple sample preparation method seem the important issues of SERS detection. Therefore, the development of quick, sensitive and reproducible on-site methods for the detection of melamine in animal feed is yet to be achieved.

SERS is a technique that can enhance the normal Raman signals from Raman-active analytes by more than 10^6^ times, sometime can even reach 10^14^ times [15], and its high sensitivity and speed of detection have attracted much attention since the 1970s [16], [17]. Du et al. detected pure melamine dissolved in 50% methanol using SERS coupled with silver-nanorod (AgNR)-array substrates, affording a limit of detection (LOD) of 0.1 µg·mL^−1^ [18]. To date, SERS has been used to detect the melamine present in food matrices such as milk, milk powder, and eggs. Liu et al. investigated the feasibility of using SERS coupled with gold substrates for the rapid detection of melamine and cyanuric acid in liquid milk, at melamine levels as high as 2 µg·mL^−1^ [19]. Cheng et al. used a portable compact Raman spectrometric system to detect the melamine adulterant in milk powder with the partial least squares analysis model [20]. They also quantified the melamine contamination in eggs using SERS with an LOD of 1.1 µg·g^−1^ [21]. The SERS detection of melamine in chicken feed has also been reported; Lin et al. detected melamine in gluten, chicken feed, and processed foods using SERS. However, the detection limits were relatively high (500 µg·g^−1^) [14].

In this study, we were able to successfully and reproducibly prepare and apply AuNPs in the SERS detection of melamine, including the development of a convenient sample preparation by liquid-liquid extraction. The method demonstrated a LOD of 0.5 µg·g^−1^, which is well within the limits established by the U.S. Food and Drug Administration, the European Union, and agencies in other countries for the determination of melamine in animal feeds.

## Materials and Methods

### Materials and apparatus

Melamine was purchased from Sigma-Aldrich Co., Ltd. (Shanghai, China). Chloroauric acid tetrahydrate (HAuCl_4_·4H_2_O), sodium hydroxide, *n*-butyl alcohol, and ethyl acetate were analytical grade and obtained from the Sinopharm Chemical Reagent Co., Ltd. (Beijing, China). The solvent mixture was prepared from *n*-butyl alcohol and ethyl acetate (6∶1 v/v). Stock solutions of melamine (1 mg·mL^−1^), stored in the dark at −18°C for 3 months prior to analysis, and their subsequent dilutions (2, 4, 6, 8, 10, and 20 µg·mL^−1^) were prepared in deionized water (18.2 MΩ·cm) for SERS analysis. All solutions were allowed to reach room temperature 2 h prior to use.

Five types of commercial feeds (formulated, complete, concentrated, mixed, and compound premix feeds) were obtained locally (collected by the Chinese National Feed Supervision in 2012 and 2014) and ground to 40-mesh powders.

A portable Raman spectrophotometer (RamTracer-200-HS) was purchased from OptoTrace Technologies, Inc. and used to collect Raman spectra. The spectral data were processed with RamanAnalyzer software, also from OptoTrace. The excitation source was a diode laser operating at 785 nm with a scanning range of 250–2500 cm^−1^ and a spectral resolution of 4 cm^−1^. The Raman scattering signals were collected by a CCD array detector. Sample glass vials (12×32×10 mm^3^) were obtained from Tegent Co., Ltd. (Guangdong, China). Measurements were conducted over the full wavelength range with a 10 s exposure time and 200 mW laser power.

A portable centrifuge (DAIHAN WiseSpin CF-10, South Korea) was used for the liquid-liquid extraction steps. Twelve centrifuge tubes could be operated simultaneously at 13500 rpm.

Transmission electron microscopy (TEM) observations were performed using a Hitachi H-7500 transmission electron microscope equipped with a Gatan 832 CCD.

### Preparation of gold nanoparticles

The synthesis of the AuNPs was based on published methods [22] with little modification. We varied the ratios of HAuCl_4_ to H_2_O and aqueous sodium citrate, the heating temperature, the stirring rate of the synthesis system, and the addition rate of the aqueous sodium citrate. Typically, all the glassware used in the procedure was dipped in aqua regia (HCl/HNO_3_, 3∶1 v/v) for 1 h, thoroughly rinsed with doubly distilled water, and then dried in a vacuum dryer at 60°C. Next, aqueous HAuCl_4_ (500 mL, 1% w/w) solution was heated to reflux with vigorous stirring (at a rate of 800 rpm), and aqueous sodium citrate (300 µL 1% w/w) was added within 2 s. The color of the solution changed from pale yellow to wine red within 1 min. The solution was heated under reflux for another 10 min. Then, the heating source was removed and the solution was continuously stirred for approximately 10 min until it had cooled to room temperature. The resultant AuNPs were stored at 4°C for no longer than 4 wk. Before using the AuNPs, they were gently vortexed for 1 min. TEM was utilized to characterize the sizes and shapes of the synthesized AuNPs.

### Sample pretreatment

**Figure 1:**
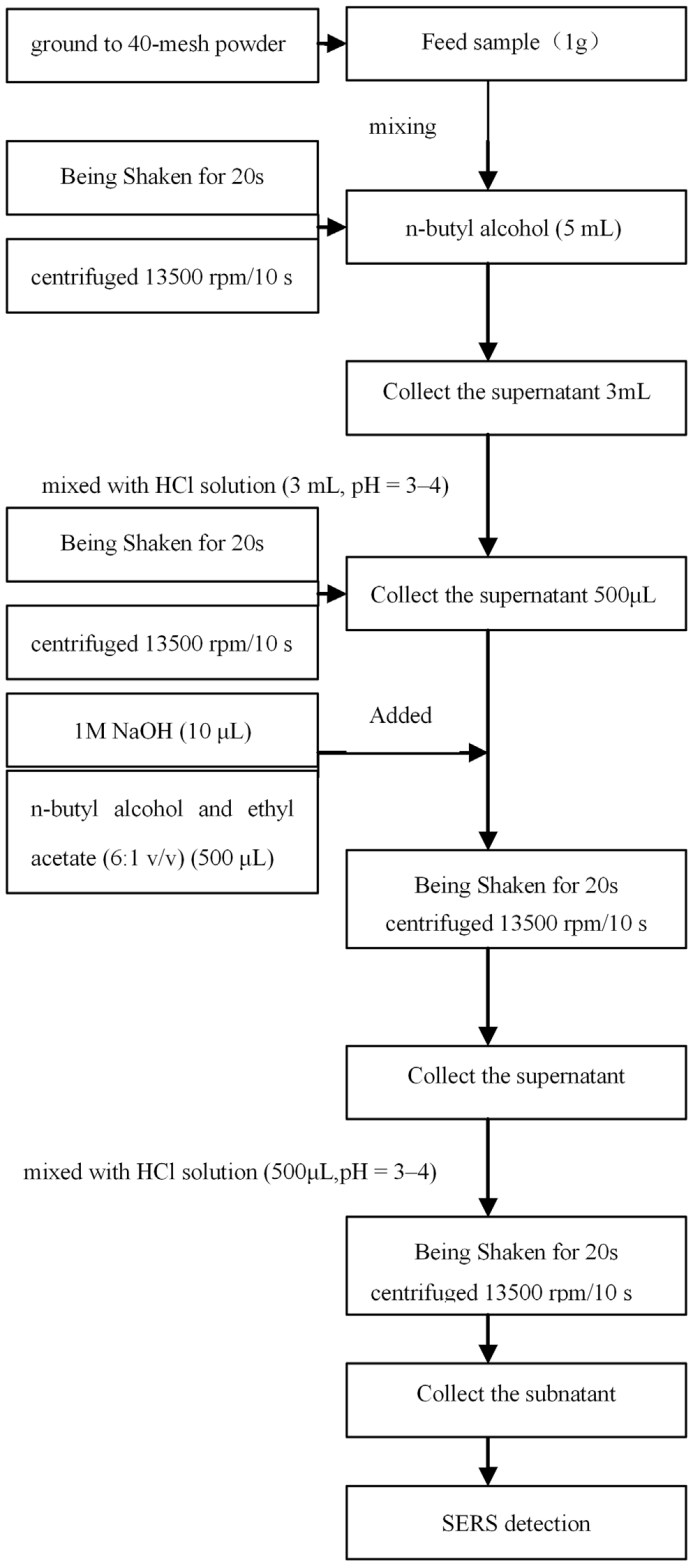
Sample pretreatment procedure flow diagram.

The experimental feed samples were spiked by mixing the blank commercially available animal feeds (formulated, complete, concentrated, mixed, and compound premix feeds) and melamine. Briefly, the feed (1 g) was spiked with melamine stock solution (0.5 µL, 1 mg·mL^−1^), and then *n*-butyl alcohol (5 mL) was added. After being shaken for 20 s, the mixture was centrifuged at 13500 rpm for 10 s. Next, 3 mL of the supernatant was mixed with HCl solution (3 mL, pH = 3–4), shaken for 20 s, and centrifuged at 13500 rpm for 10 s. Then, 500 µL of the subnatant was added into 1 M NaOH (10 µL) and the solvent mixture (500 µL), which was then shaken for 20 s and centrifuged at 13500 rpm for 10 s. The supernatant so obtained was mixed with HCl solution (500 µL), shaken for 20 s, and centrifuged at 13500 rpm for another 10 s. Finally, the subnatant obtained at the end of the process was used for SERS detection. The sample pretreatment procedure flow diagram is shown in Fig. 1.

### SERS measurement

In this step, the final subnatant (30 µL) was transferred to a glass vial, into which the AuNPs solution (500 µL) and a 1% inorganic salt solution (50 µL) were added. The inorganic salt was typically chloride ion-containing (e.g., sodium chloride or calcium chloride). After vortexing gently for 10 s, the mixed nanoparticle solution was detected over the scanning range 250–2500 cm^−1^ with the portable Raman spectrophotometer, at 200 mW with an exposure time of 10 s. The spectral resolution was set to 4 cm^−1^ and each spectrum consisted of two scans.

### Data Analysis

The SERS spectral data were analyzed by the RamanAnalyzer software from 500 to 2500 cm^−1^. The baseline shift was offset using the Savitzky–Golay second derivative transformation [23], and pre-processing algorithms such as polynomial subtraction and smoothing were employed to analyze the data. To eliminate the effects of the matrix and other factors such as temperature, humidity, and focal distance, the intensity of the peak observed in the blank feed at 731 cm^−1^ was first set to 100 as a basis for the samples, and then the Raman peak intensity of melamine at 707 cm^−1^ was calculated.

## Results and Discussion

### Characterization of AuNPs

**Figure 2:**
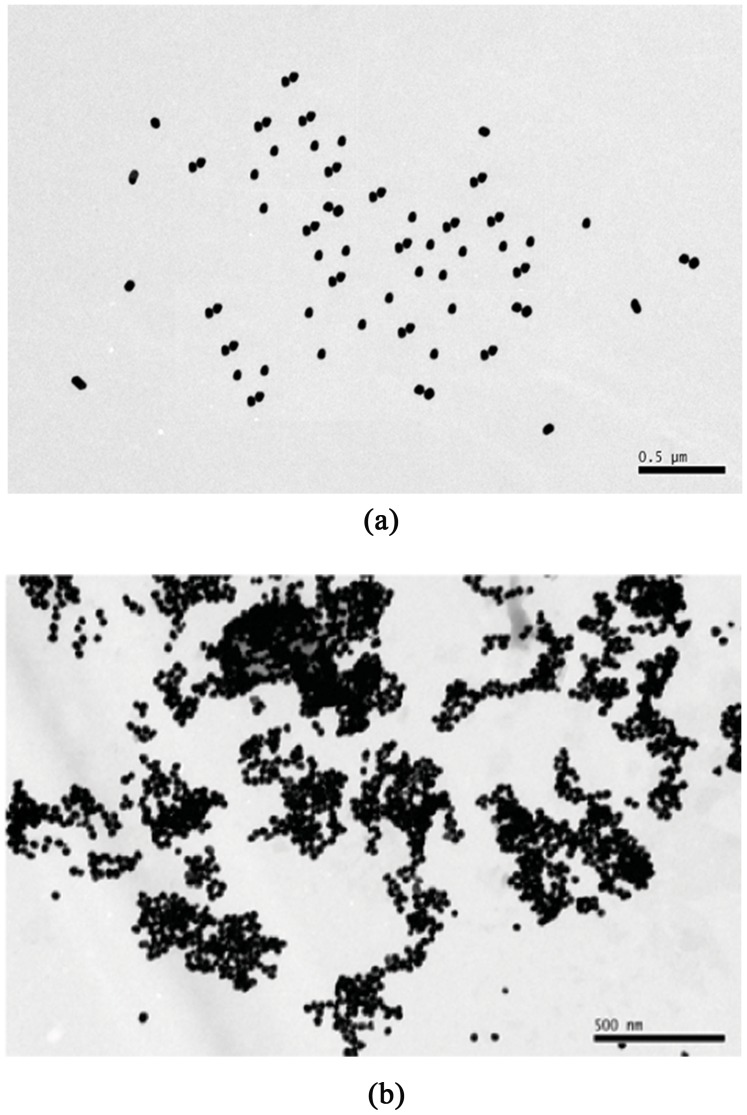
Enhancement of SERS sensitivity after AuNP aggregation. TEM images of (a) synthesized AuNPs and (b) synthesized AuNPs after addition of 50 µL 1% inorganic salt solution.

To date, much attention has been paid to the adsorption properties of AuNPs, which are major factors influencing the Raman signal enhancement in the SERS technique. In general, nanoscale adsorption is strongly influenced by chloride ions [24] and pH [25]. TEM images of the AuNPs are shown in Figs. 2(a) and (b). As seen in Fig. 2(a), the diameters of the AuNPs were approximately 55 nm, but after adding 1% NaCl (50 µL), the AuNPs formed inhomogeneous aggregates (Fig. 2(b)) that provided the most optimal form for the enhancement of SERS. Therefore, this system was chosen as the substrate for our study. Figs. S1(a) and S1(b) show the SERS spectra of 1 µg·mL^−1^ melamine on the AuNP substrates from Figs. 2(a) and 2(b) (before and after aggregation), respectively. It is well known [26], [27], [28] that the addition of chloride ions, for example, to a given colloid can enhance the SERS signal by a factor of more than 10. A widely accepted model explains this observation with the postulate that the chloride ions are necessary to mediate the binding of the adsorbate to the surface or, alternatively, to ensure an adsorption geometry which gives rise to a large enhancement factor (chemical effect) [27], [29]. And the addition of chloride ions may induced the large increases in the electric field near the surface (electro-magnetic effect) because of the improved aggregation of AuNPs which is also responsible for a large enhancement factor [22], [30].

### Characteristic peak of melamine

**Figure 3:**
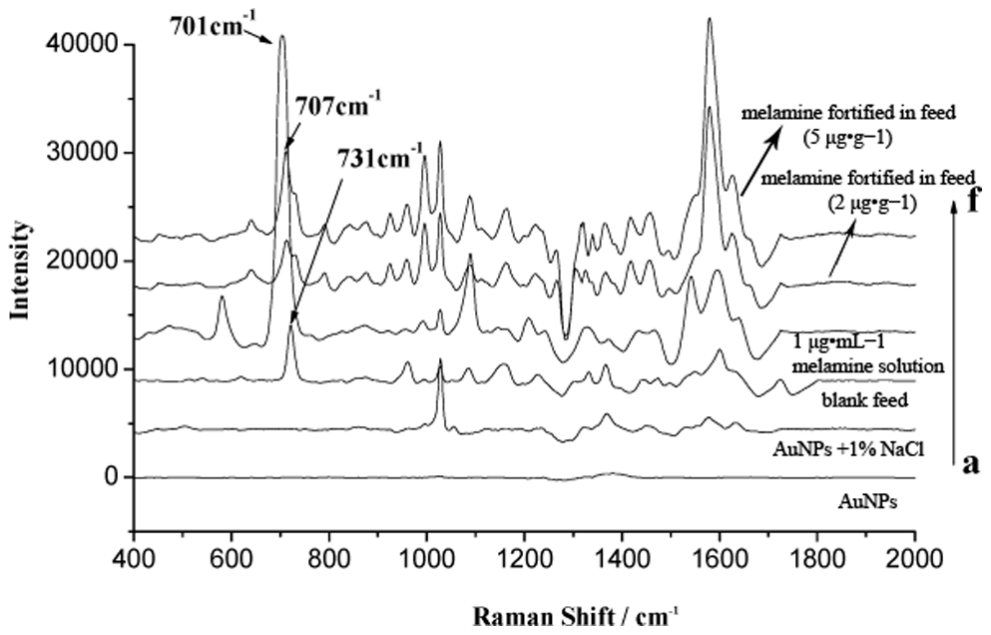
SERS spectra: blanks, standards, and representative samples. The SERS spectra of (a) synthesized AuNPs; (b) synthesized AuNPs after the addition of 1% NaCl; (c) blank feed (after sample treatment); (d) melamine standard solution (1 µg·mL^−1^); (e) feed sample spiked with melamine (2 µg·g^−1^); and (f) feed sample spiked with melamine (5 µg·g^−1^).

To find the characteristic peaks of melamine, we investigated the Raman spectra of the AuNPs, AuNPs +1% NaCl, blank feed (after sample treatment), melamine standard solution (1 µg·mL^−1^), and blank feed (after sample treatment) spiked with a standard melamine solution (spiked concentration, 2 µg·g^−1^). The most intense Raman peak associated with the melamine standard solution was observed at 701 cm^−1^ (Fig. 3(d)). In the actual feed samples, the characteristic peak shifted from 701 to 707 cm^−1^ (Fig. 3(e,f)), which may be caused by matrix effects. In the spectra of the AuNPs, AuNPs +1% NaCl, and blank feed (Fig. 3(a–c)), the peak at 707 cm^−1^ was not observed. We concluded that the peak at 707 cm^−1^ was the ideal peak characteristic of melamine in the feed matrices to use for detection/quantification. In addition, to eliminate the effects of the matrix and other factors such as temperature, humidity, and focal distance, the intensity of the peak observed in the blank feed at 731 cm^−1^ was first set at 100 as a basis for the samples, and then the Raman peak intensity at 707 cm^−1^ was calculated using RamanAnalyzer software.

**Figure 4:**
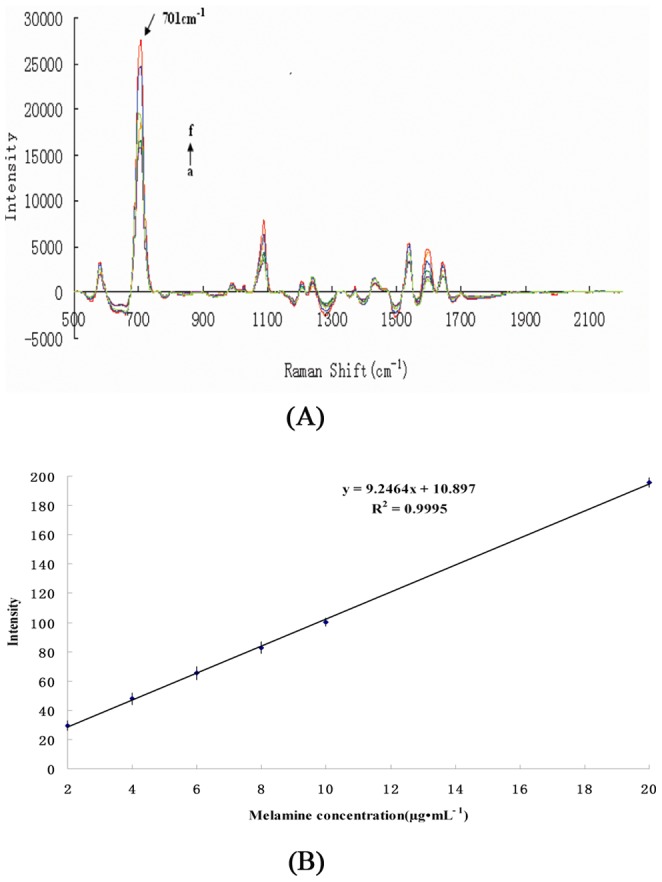
Establishment of SERS standard curves. (A)SERS spectra for different concentrations of melamine standard solution: (a) 2, (b) 4, (c) 6, (d) 8, (e) 10, and (f) 20 µg·mL^−1^. (B) Melamine concentration–peak intensity curve for standard solutions in the range 2–20 µg·mL^−1^.

The SERS spectra for different concentrations of melamine standard solution (2, 4, 6, 8, 10, and 20 µg·mL^−1^) (n = 6) are shown in Fig. 4(A). As the concentration of melamine increases, the signal intensity of the characteristic peak also increases. A linear regression (*R* ^2^ = 0.999) was applied to the relationship between the Raman peak intensity at 701 cm^−1^ and the melamine concentration in the standard solutions (Fig. 4(B)). The peak at 701 cm^−1^ is assigned to the ring-breathing II mode, which involves the in-plane deformation of the triazine ring.

### Optimization of sample treatment

#### Interference

**Figure 5:**
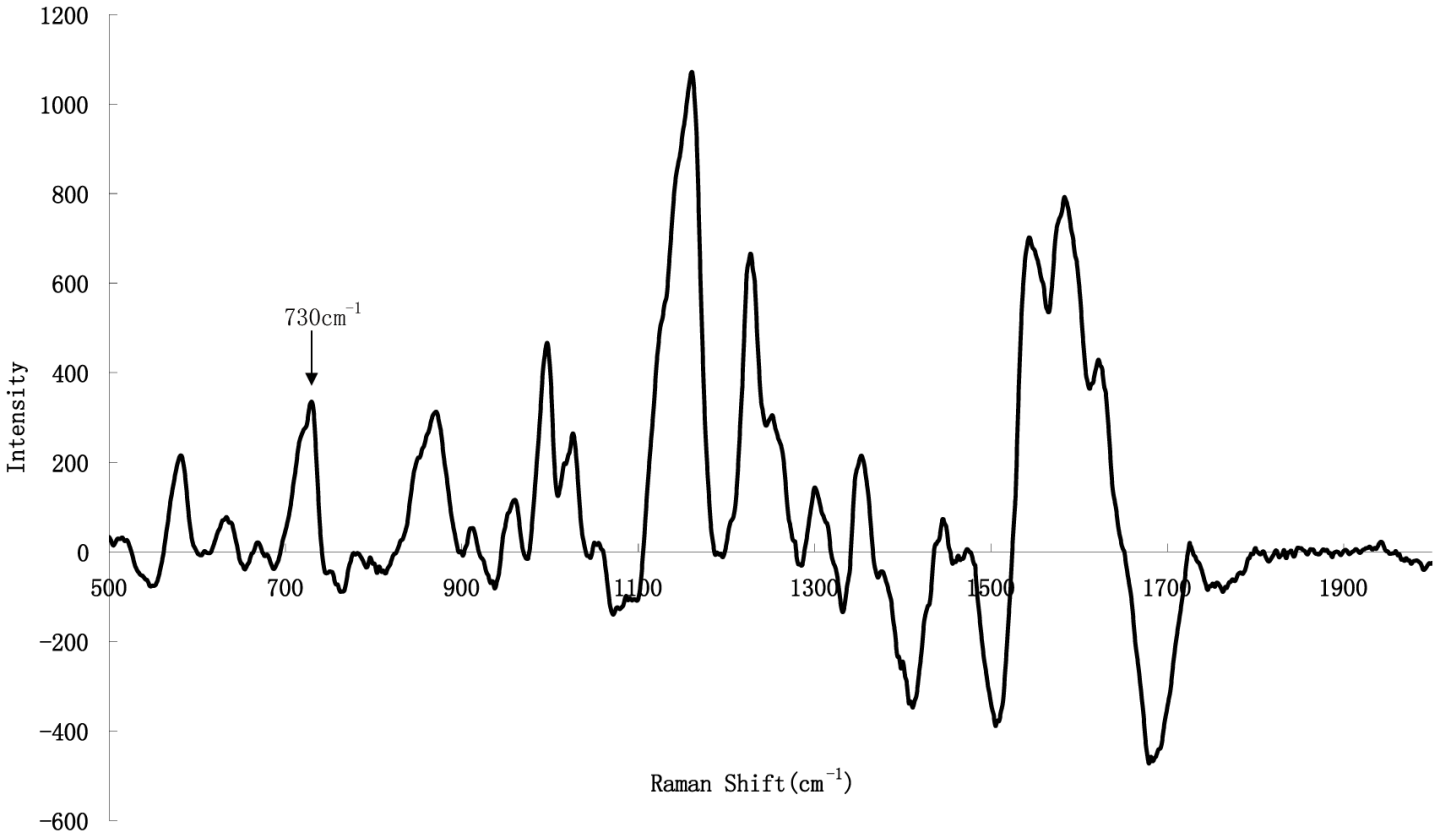
SERS spectrum of a feed sample extract spiked with melamine (10 µg·mL^−1^) using trichloroacetic acid as extraction solvent and double liquid-liquid extraction pretreatment.

Feed samples are complex matrices that are difficult to analyze mainly because of their protein content, which introduces the most interference in melamine analysis. Figure 5 shows the Raman spectrum of a solution extracted from a feed sample and spiked with 10 µg·mL^−1^ melamine. In this figure, the characteristic melamine Raman shift at 707 cm^−1^ is obscured due to overlap with a large matrix-based peak at 731 cm^−1^. In this study, we optimized the sample treatment method (as described above) to remove most of the interference from the feed sample.

#### Impact of extraction solution and optimization of clean-up

**Figure 6:**
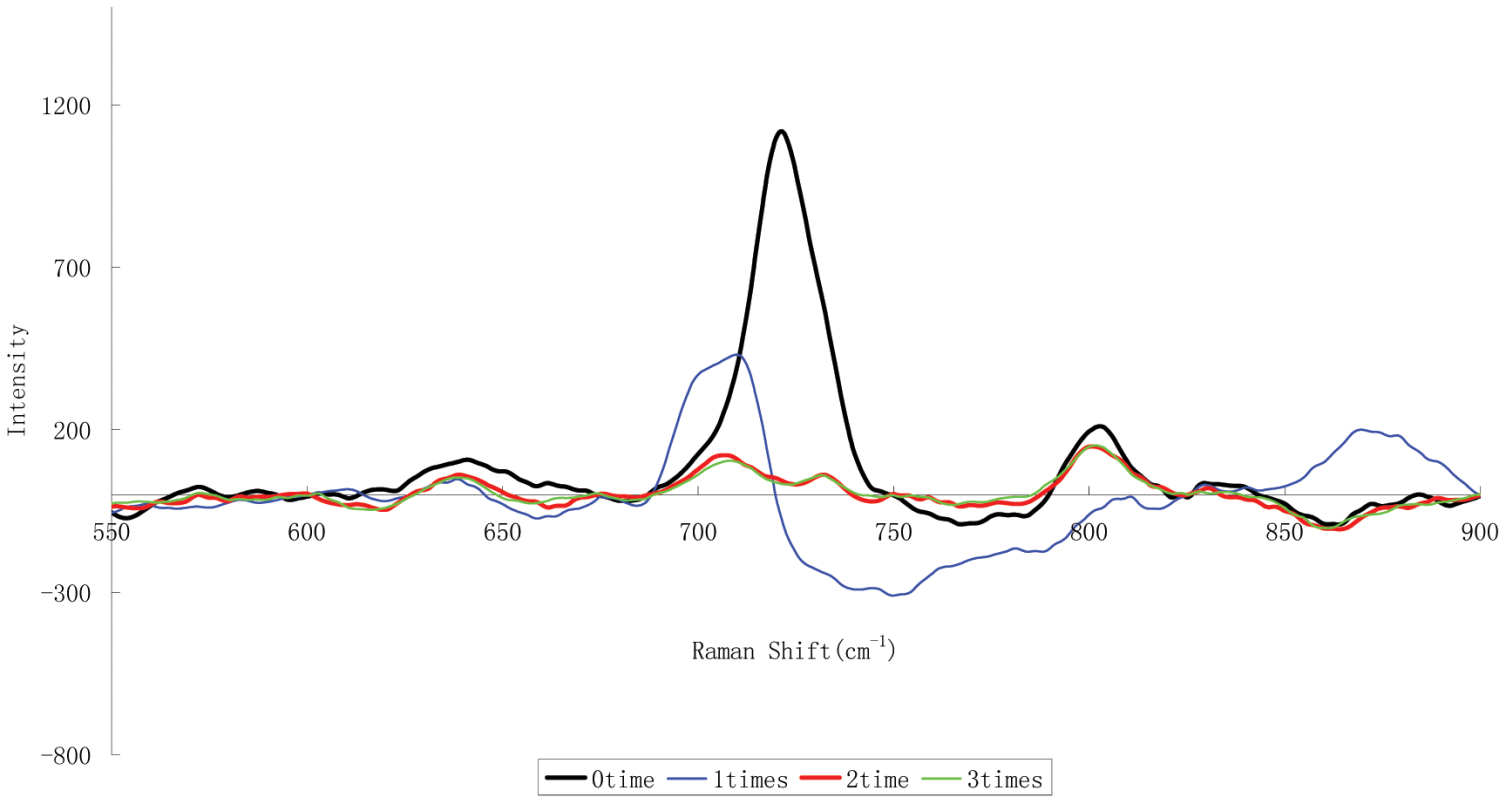
SERS spectra of samples pretreated by liquid-liquid extraction per number of extraction cycles.

The extraction of melamine from feed samples is critical to its determination, and has been carried out using 50% (v/v) acetonitrile in water [14] or an acid solution such as trichloroacetic acid, acetic acid, hydrochloric acid, or trifluoroacetic acid [31]. However, these extraction methods are not appropriate for the SERS detection in this study because the peak at 731 cm^−1^ would overlap with the characteristic peak of melamine (shown in Fig. 5). *n*-Butyl alcohol was chosen as the extraction solvent, considering its solubilization and selective extraction of melamine away from matrix interference and its potential to remove interfering compounds such as free amino acids, protein molecules, and pigments. Based on the pK_a_ of melamine, liquid–liquid extractions were performed twice using HCl (pH 3–4) and a solvent mixture (*n*-butyl alcohol/ethyl acetate, 6∶1 v/v). When we added HCl, the protonation of melamine allowed its extraction from butyl alcohol into the aqueous phase, partially removing proteins and pigments. In the second step, the aqueous phase was made alkaline (pH = 9–10) with 1 M NaOH, which neutralized the melamine salt but did not lead to further ionization. Thus, we were able to extract the melamine from the aqueous phase into 6∶1 (v/v) *n*-butyl alcohol/ethyl acetate while simultaneously separating it from the polar compounds. Two iterations of this liquid–liquid extraction process achieved the most effective purification (Fig. 6). This method sufficiently resolved the peaks at 731 and 707 cm^−1^ and did not negatively affect the qualitative and quantitative analyses of melamine. Moreover, this sample pretreatment method does not require a solid-phase-extraction operation. The average sample analysis time was approximately 5 min.

### Validation of proposed method

**Figure 7:**
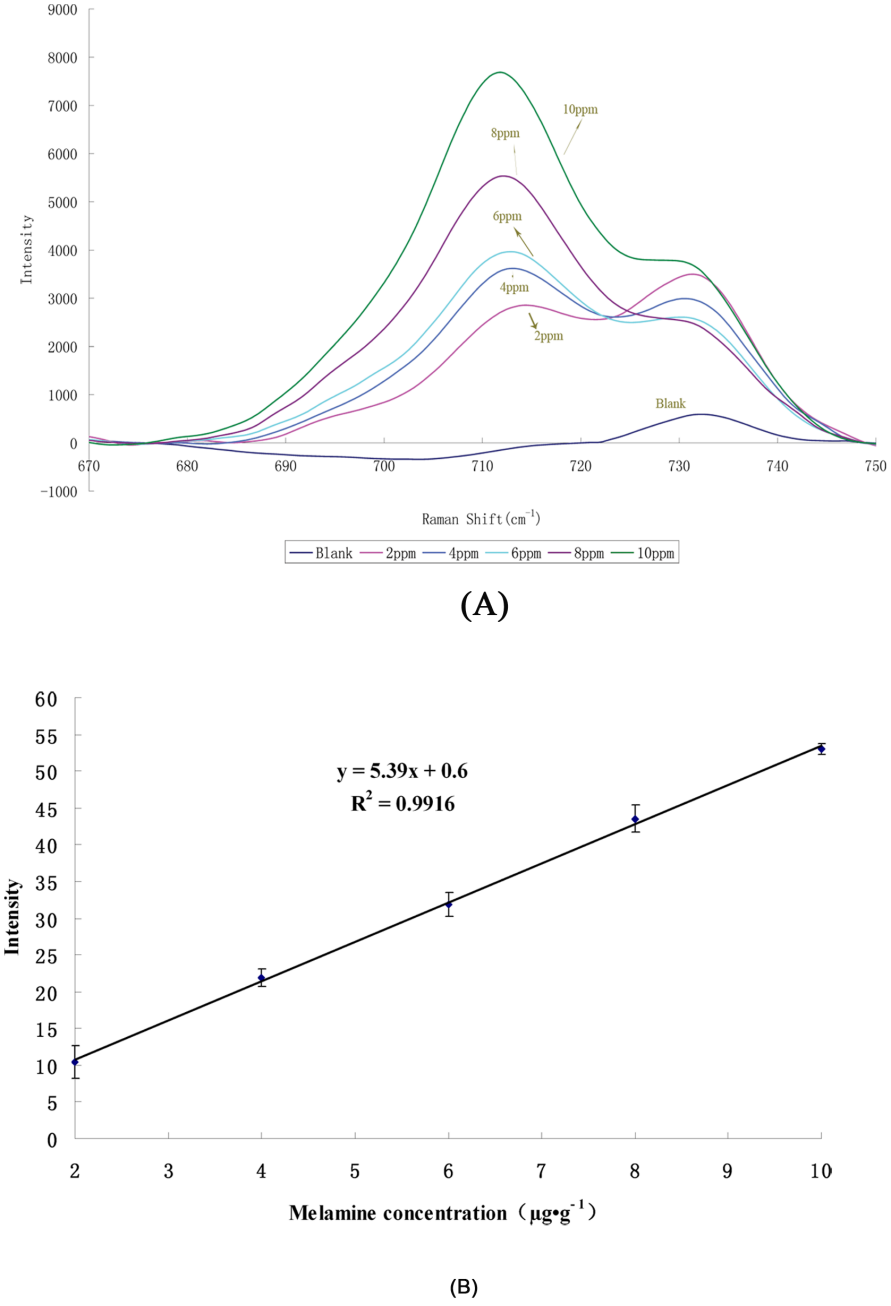
(A)SERS spectra of different melamine concentrations in spiked feed samples: (a) blank, (b) 2, (c) 4, (d) 6, (e) 8, and (f) 10 µg·g^−1^ (**B**) Melamine concentration fortified in feed−response curve for feed samples spiked with melamine in the range 2–10 µg·g^−1^.

To investigate the effect of the matrix on the proposed method, feed samples spiked with known concentrations of melamine (0, 2, 4, 6, 8, and 10 µg·g^−1^) (n = 6) were analyzed. The resulting SERS spectra are shown in Fig. 7(A). A linear regression analysis (R^2^ = 0.9916) was applied to the relationship between the Raman peak intensity at 707 cm^−1^ and the different concentrations of melamine in the fortified feed (Fig. 7(B)) for the range 2–10 µg·g^−1^. The peak intensity was saturated when the melamine concentration was greater than 10 µg·g^−1^.

When the calibration graphs obtained from the model melamine solutions and melamine-spiked feed samples were compared, it was found that slopes of the calibration curves were very different, with values of 9.2464 for the model melamine solution and 5.39 for the melamine-spiked feed (Fig. 4(B) and 7(B)). This indicates the method is affected by feed-based interference. To eliminate the effect of the matrix in the proposed method, matrix-matched calibration standard curves were selected to quantify melamine in the feed samples.

During analysis, if the response exceeded the linear range, the sample was diluted appropriately and re-tested. The high value of the coefficient of linearity shows that the method is not significantly affected by feed-based interference.

The limit of detection (LOD) of feed substrates for melamine samples is defined as the lowest concentration at which a positive melamine SERS spectrum can be obtained. In our experiment, the LOD is the lowest concentration at which the peak intensity of the feed samples at 707 cm^−1^ is significantly different from the blank feed (control samples) which was detected by GC-MS method. At least 9 spectra from two different blank feed substrates were measured. The peak intensity at 707 cm^−1^ of blank feed were recorded. The value of average plus three times the standard deviation of the blank feed was set as the limit for determining a positive detecton from a negative detection [32]. This means that when the sample spectrum intensity at 707 cm^−1^ has a greater value than the limit values above, the sample is the positive one. And the limit of quantification (LOQ) is defined by the similar method, which was calculated by the value of average plus ten times the standard deviation of the blank feed. In our experiment, we analysed blank samples spiked at different concentrations (decreased the spiked concentration step by step) and validated the sample pretreatment method and the response of the instrument. After adjusting the concentration, the lowest concentration that yield all positive detections (LOD) was approximately 0.5 µg·g^−1^ and 2 µg·g^−1^ was the limit of quantification. The LOD and LOQ values in the feed fall below the maximum residual limit (2.5 µg·g^−1^) established by the European Union. The linear range is sufficient for the quantification of melamine in accordance with legal limits.

Melamine recoveries were determined for feed samples spiked with 2, 6, 8, and 10 µg·g^−1^ of melamine and were found to be 83.5, 90.2, 88.6, and 82.5%, respectively (Table 1). These results indicate the reliability of the method used for melamine detection in feed samples. The results are acceptable according to EU recommendations that establish the range as 80–110% for concentrations of 10 µg·g^−1^ [7].

**Table 1:** Recovery of melamine from spiked feed samples.

Concentration of melamine spiked in feed samples (µg·g^−1^)	Average intensity^a^ of the peak at 707 cm^−1^	Amount found^b^ (µg·g^−1^)	Recovery (%) (n = 5)	RSD^c^ (%)
2	12.2	1.67±0.09	83.5	4.02
6	30.4	5.41±0.02	90.2	0.82
8	43.3	7.09±0.05	88.6	0.91
10	55.9	8.25±0.07	82.5	2.07

a Intensity values at 707 cm^−1^ are calculated after the peak at 731 cm^−1^ is set to 100.

b Average values from six determinations for each concentration.

c Relative standard deviation of peak intensity (RSD (%)  =  (SD/mean) ×100).

The intraday and interday precisions of the method were determined for feed samples spiked with 5 µg·g^−1^ melamine (Table 2), and precision was described using the relative standard deviation (RSD). The repeatability of the results is expressed as the RSDs of the intraday and interday measurements; the RSD values ranged from 0.97 to 1.98%, respectively. Thus, it can be concluded that the method had good repeatability for the detection of melamine in feeds.

**Table 2:** Interday and intraday repeatability of the method.

Analysis^a^	Measurements	Spiked concentration (µg·g^−1^)	Amount found^b^ (µg·g^−1^)	Average intensity^a^ of the peak at 707 cm^−1^	Precision as RSD^b^ (%)	RSD^c^ (%)
Intraday	Day 1	5	4.89±0.14	28.16	6.5	0.97
	Day 1	5	4.95±0.05	28.38	3.8	
	Day 1	5	4.97±0.04	28.76	3.3	
	Day 1	5	5.02±0.09	29.09	5.8	
	Day 1	5	4.93±0.03	28.27	2.8	
Interday	Day 1	5	4.91±0.11	28.10	6.2	1.98
	Day 2	5	4.82±0.08	28.01	5.6	
	Day 3	5	4.98±0.05	28.90	3.8	
	Day 4	5	4.85±0.12	28.04	6.3	
	Day 5	5	5.06±0.07	29.20	5.4	

a Average of three determinations for each measurement.

b Relative standard deviation (RSD (%)  =  (SD/mean) ×100) of individual measurements or days.

c RSD % of intraday and interday measurements.

### Application of the proposed method

In this study, the SERS method was precise and could be applied in the field detection of melamine in the various feed samples (i.e., the formulated, complete, concentrated, mixed, and compound premixed feeds). Forty samples of the five types of listed feeds, obtained from the Chinese National Feed Supervision from 2012 to 2014, were tested by GC/MS [33] and SERS. The test results from the SERS method were validated and corroborated by GC/MS (Table 3) for all 40 samples.

**Table 3:** Comparison of results obtained by GC/MS and SERS in 40 feed samples.

Sample Number	GC/MS (µg·g^−1^)	SERS	
		Quantity (µg·g^−1^)	Quality^b^
BCT20120175	0.13	ND	Negative
BCT20120187	ND^a^	ND	Negative
BCT20120190	ND	ND	Negative
BCT20120191	ND	ND	Negative
BCT20121167	ND	ND	Negative
BCT20121365	ND	ND	Negative
BCT20122189	ND	ND	Negative
BCT20122190	10.78	11.23	Positive
BCT20122230	ND	ND	Negative
BCT20122470	ND	ND	Negative
BCT20130024	ND	ND	Negative
BCT20130037	ND	ND	Negative
BCT20130098	44.38	46.33	Positive
BCT20130120	0.78	ND	Negative
BCT20130123	ND	ND	Negative
BCT20130124	ND	ND	Negative
BCT20130154	ND	ND	Negative
BCT20130167	ND	ND	Negative
BCT20130169	ND	ND	Negative
BCT20130180	ND	ND	Negative
BCT20130205	ND	ND	Negative
BCT20130207	ND	ND	Negative
BCT20130229	ND	ND	Negative
BCT20130291	ND	ND	Negative
BCT20130301	19.61	20.98	Positive
BCT20130322	12.09	13.22	Positive
BCT20130341	ND	ND	Negative
BCT20130342	ND	ND	Negative
BCT20130343	ND	ND	Negative
BCT20130344	ND	ND	Negative
BCT20130358	68.90	70.01	Positive
BCT20130366	ND	ND	Negative
BCT20140008	6.70	7.52	Positive
BCT20140020	ND	ND	Negative
BCT20140023	ND	ND	Negative
BCT20140024	ND	ND	Negative
BCT20140034	ND	ND	Negative
BCT20140050	ND	ND	Negative
BCT20140053	ND	ND	Negative
BCT20140180	ND	ND	Negative

a Not detected means the melamine content was below the LOD of the method (0.05 µg·g^−1^).

b A negative result means the content of melamine in the sample was below a concentration of 2.5 µg·g^−1^.

In China, the government currently surveys the melamine content in feeds using the GC/MS method. Compared with this method, the SERS technique requires relatively simple sample preparation because no complex clean-up operations are needed. Additionally, the GC/MS method requires derivation that consumes approximately 30 min/sample. The total detection time using SERS is approximately 5 min/sample.

## Conclusions

SERS is a novel emerging analytical technique with satisfactory performance in the detection of trace amounts of analytes. In this study, a rapid, selective, and sensitive SERS method based on the aggregation of 55 nm AuNPs was developed. This new protocol could be developed for the routine on-site monitoring of feeds, for the purposes of quality control and market surveillance.

## Supporting Information

Figure S1 **SERS spectra of 1 µg·mL^−1^ melamine (a) on the synthesized AuNPs and (b) on the enhanced, inhomogeneously aggregated AuNPs.** The unaggregated AuNPs (a) show a weak Raman signal characteristic of melamine at 707 cm^−1^. After the inorganic salt solution was added, the characteristic melamine signal was strongly enhanced (b).
(TIF)

Figure S2 **The different storage conditions versus the intensity of the melamine Raman shift at 701 cm^−1^.**
(TIF)

Figure S3 **The different pre-use vortexing times (at 1250 rpm for 0.5, 1, 1.5, 2, and 2.5 min) of the AuNPs versus the intensity of the melamine Raman shift at 701 cm^−1^.**
(TIF)

Figure S4 **The effect of different vortexing times of the sample mixtures (at 1250 rpm for 2, 5, 10, 15, and 20 s) on the SERS intensity at 701 cm^−1^.**
(TIF)

Figure S5 **Effects of vial cleaning solvent.** The intensity of the characteristic signal at 701 cm^−1^ decreased rapidly after each cleaning.
(TIF)

Text S1 **Reproducibility in the SERS substrates.**
(DOCX)
