# Supplementary figures and images for: Detection of Melamine in Feed Using Liquid-Liquid Extraction Treatment Combined with Surface-Enhanced Raman Scattering Spectroscopy
Source: PLoS One. 2014 Sep 22;9(9):e107770. doi: 10.1371/journal.pone.0107770 (PMC4171497; doi:10.1371/journal.pone.0107770)

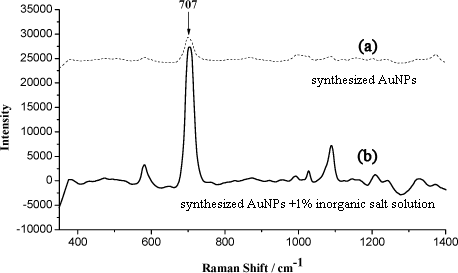

Supplement: Figure S1 — SERS spectra of 1 µg·mL−1 melamine (a) on the synthesized AuNPs and (b) on the enhanced, inhomogeneously aggregated AuNPs. The unaggregated AuNPs (a) show a weak Raman signal characteristic of melamine at 707 cm−1. After the inorganic salt solution was added, the characteristic melamine signal was strongly enhanced (b). (TIF) [file pone.0107770.s001.tif]

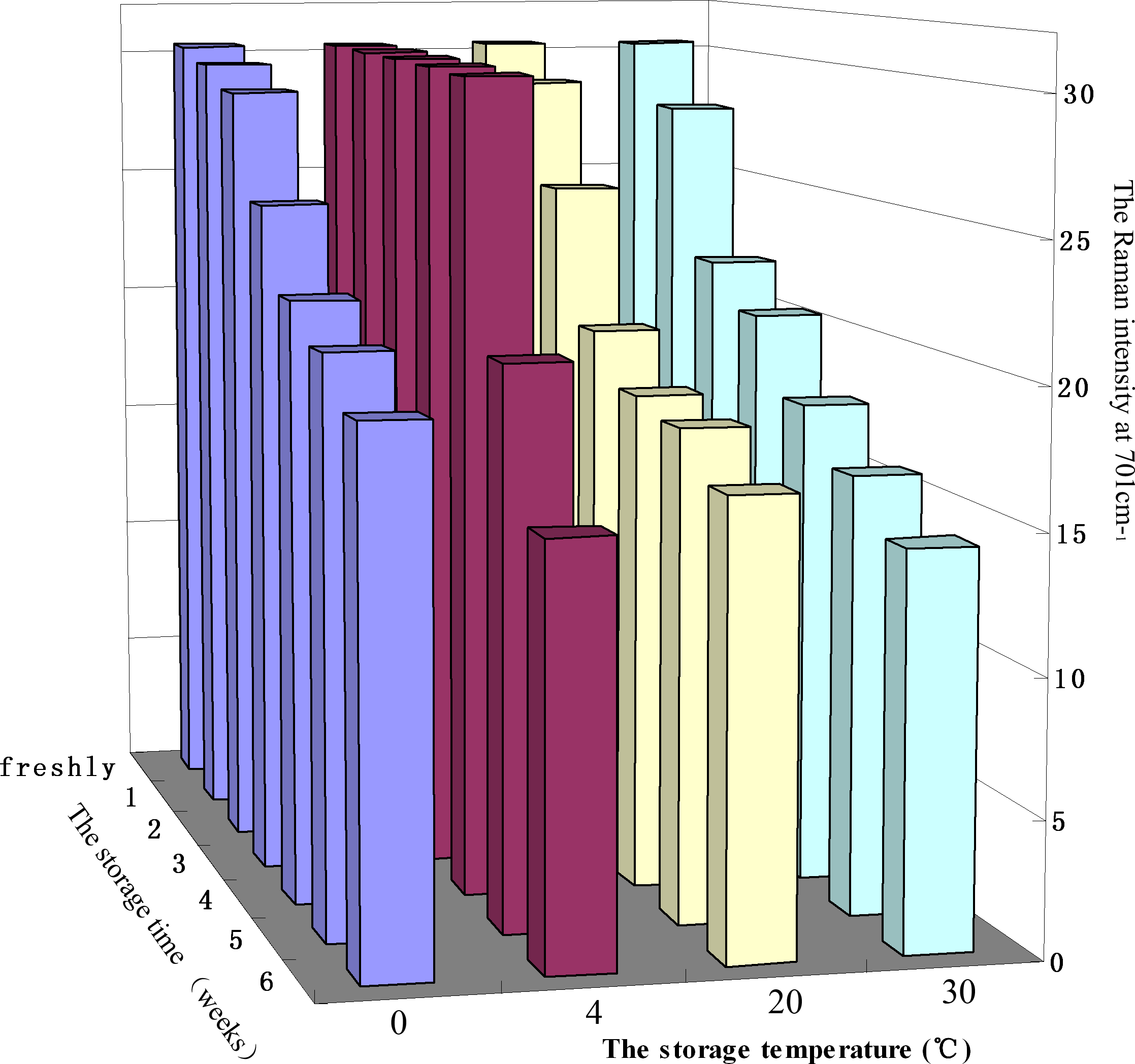

Supplement: Figure S2 — The different storage conditions versus the intensity of the melamine Raman shift at 701 cm−1. (TIF) [file pone.0107770.s002.tif]

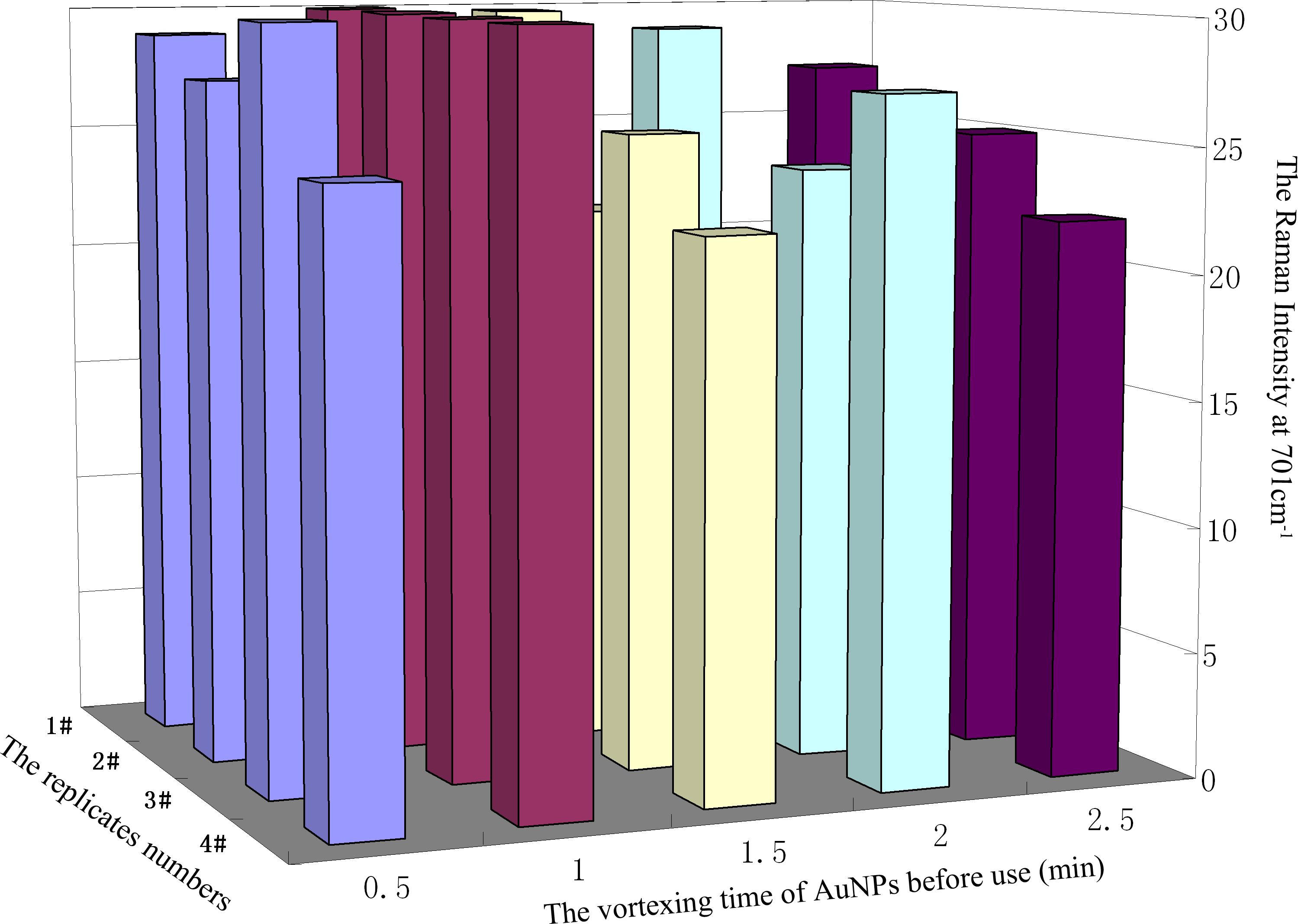

Supplement: Figure S3 — The different pre-use vortexing times (at 1250 rpm for 0.5, 1, 1.5, 2, and 2.5 min) of the AuNPs versus the intensity of the melamine Raman shift at 701 cm−1. (TIF) [file pone.0107770.s003.tif]

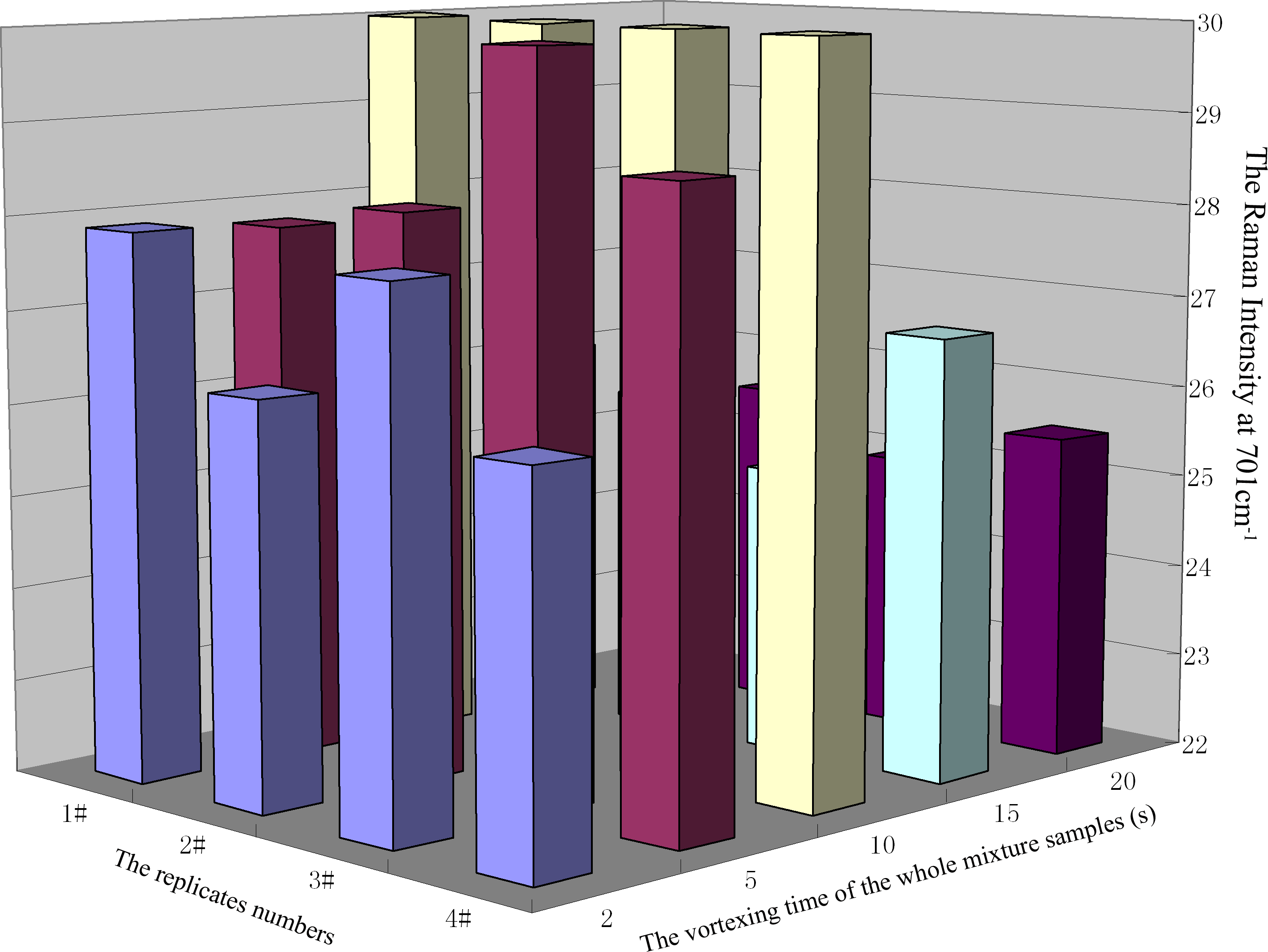

Supplement: Figure S4 — The effect of different vortexing times of the sample mixtures (at 1250 rpm for 2, 5, 10, 15, and 20 s) on the SERS intensity at 701 cm−1. (TIF) [file pone.0107770.s004.tif]

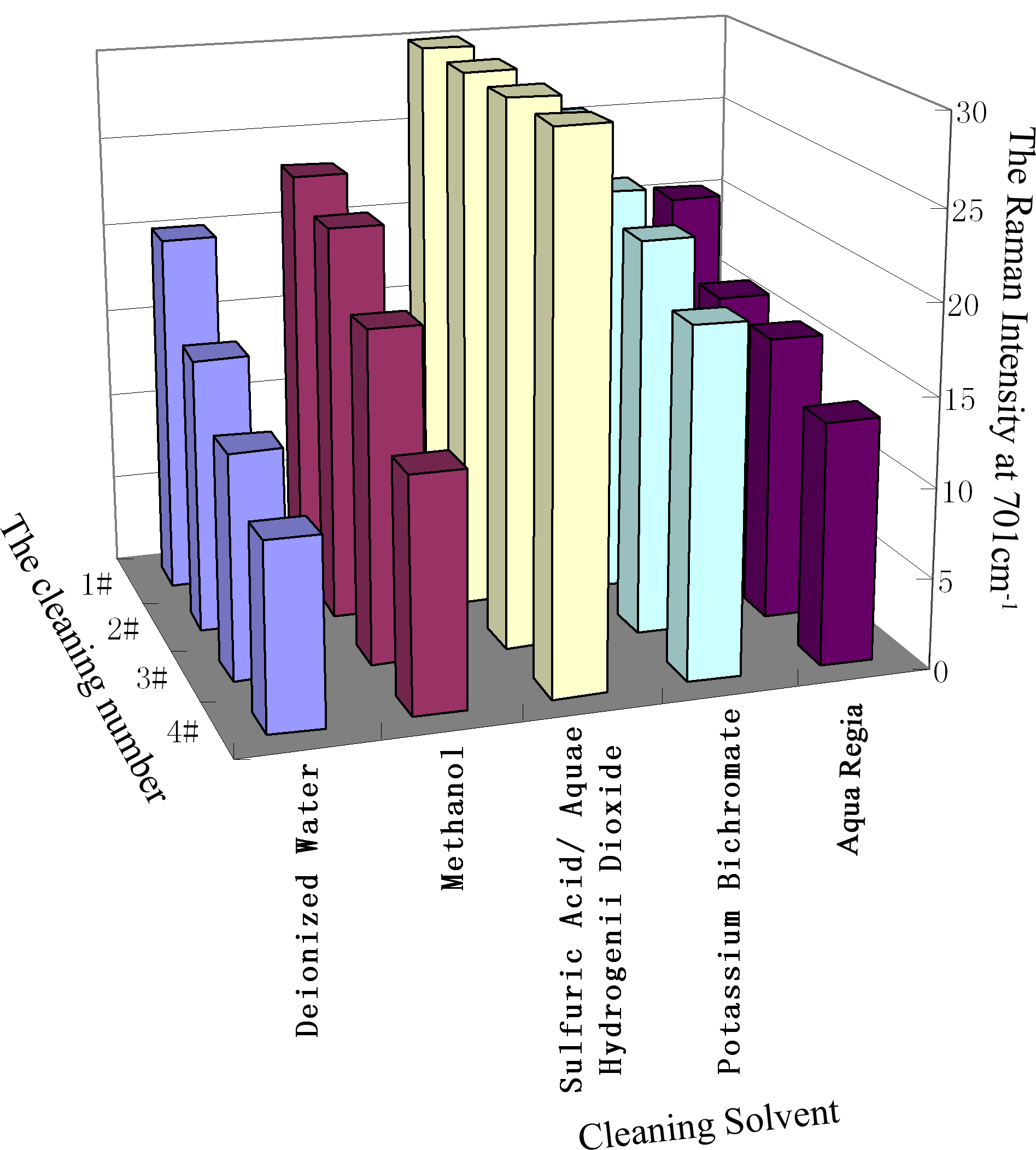

Supplement: Figure S5 — Effects of vial cleaning solvent. The intensity of the characteristic signal at 701 cm−1 decreased rapidly after each cleaning. (TIF) [file pone.0107770.s005.tif]
