# Supplementary material for: Detection of Melamine in Feed Using Liquid-Liquid Extraction Treatment Combined with Surface-Enhanced Raman Scattering Spectroscopy
Source: PLoS One. 2014 Sep 22;9(9):e107770. doi: 10.1371/journal.pone.0107770 (PMC4171497; doi:10.1371/journal.pone.0107770)
Supplement: Text S1 — Reproducibility in the SERS substrates. (DOCX) [file pone.0107770.s006.docx]

**Supporting Information**

**Text S1.** **Reproducibility in the SERS substrates**

When colloids have been employed as SERS substrates, the issue of reproducibility has been addressed. There has been continued interest in improving the reproducibility of SERS intensity measurements. Different approaches have been used, such as improving the protocols used during the measurement stage [1, 2, 3]. Other sources of reproducibility problems, such as colloid preparation (e.g., stirring rate), have been shown to have contributory roles [4]. Furthermore, SERS measurements are complicated even more by the fact that different probe molecules require different optimized protocols for optimum SERS intensities [5].

Most SERS studies are aimed at understanding the colloid aggregation events during SERS measurement as this plays an important role in determining reproducibility. Many studies have concluded that the final size and shape of the metal clusters formed during aggregation will affect SERS enhancement [6].

In our study, the protocols for the preparation of the AuNPs were well controlled. For example, the AuNPs storage conditions, the length of time the AuNPs were vortexed before use, the length of vortexing time for sample mixing, and the cleaning of the glass bottles which were used for the SERS detection were the main factors influencing the aggregation of the AuNPs and affecting the reproducibility of the melamine-SERS intensity results.

**AuNPs storage conditions**

For the storage-temperature stability study, aliquots of freshly prepared AuNPs (25 mL) were stored in 50 mL polypropylene centrifugation tubes under four different storage conditions: 0, 4, 20, and 30°C for 1, 2, 3, 4, 5, and 6 wk. Each week, SERS measurements of the melamine standard solution (1 μg·mL^−1^) on the stored colloids were performed. The same measurement protocol for SERS analysis was used as detailed above. Six repeat measurements per storage condition were acquired each week.

Figure S2 compares the different storage conditions versus the intensity of the melamine Raman shift at 701 cm^−1^. From the figure, we determined that the optimal storage temperature was 4°C, at which the intensity of the Raman shift at 701 cm^−1^ remained relatively stable (the intensity changed from 30.33 to 30.11 over 4 wk). Storage temperatures above or below 4°C had severe effects on the intensity of the melamine Raman shift at 701 cm^−1^. As the storage time increased, the intensity of the characteristic melamine signal decreased, which may reflect the severe self-aggregation of the AuNPs. These conclusions paralleled those previously reported in the literature [7]. The acceptable storage time was less than 4 wk. After this period, the intensity of the Raman shift at 701 cm^−1^ decreased rapidly, most likely because of self-aggregation or even sedimentation of the AuNPs.

**Effects of vortexing duration before use and during mixing**

To perform the SERS analysis, the prepared AuNPs solution (500 μL, gently vortexed at 1250 rpm for 1 min) and 50 μL 1% sodium chloride (or calcium chloride) were mixed in a 2 mL glass vial. The melamine standard solution (30 μL, 1 μg·mL^−1^) was transferred into the glass vial. The whole mixture was vortexed (1250 rpm) for 10 s before SERS detection. Variations of the above procedures were performed to study the effect of the vortexing times of the AuNPs before use and of the whole sample mixture on the reproducibility of melamine-SERS intensities. Thus, we varied the durations of vortexing (1) of the AuNPs prior to use (0.5, 1, 1.5, 2, and 2.5 min); and (2) of the whole mixture before SERS detection (2, 5, 10, 15, and 20 s).

Figure S3 shows the different vortexing times (at 1250 rpm for 0.5, 1, 1.5, 2, and 2.5 min) of the AuNPs (after less than 4 wk storage at 4°C) versus the intensity of the melamine Raman shift at 701 cm^−1^. Four replicates were measured. Because of the stable signal intensity at 701 cm^−1^, an optimal vortexing time of 1 min was selected. Figure S4 shows the effects of vortexing duration for the sample mixtures (at 1250 rpm for 2, 5, 10, 15, and 20 s) on the SERS intensity at 701 cm^−1^. In this case, four replications were performed for each duration. The optimal mixing time was 10 s.

The intensity reproducibility was improved by optimizing the vortexing time; such improvements are thought to be associated with the formation of reproducible metal clusters under the conditions of forced convection, so as to reduce the effects of random collisions governed by natural convection [7]. The different storage conditions hugely affected the extent of SERS data reproducibility. These two conclusions agreed with those reported in a previous study [7].

**Glass vial cleaning protocol**

After the tests, the glass vials must be washed and dried in preparation for the next use. The selection of the cleaning solvent was quite important, as it affected the reproducibility of the intensity. We washed glass vials 4 times. After each wash time, SERS measurements of the melamine standard solution (1 μg·mL^−1^) on the same washed glass vials were performed. Common cleaning solvents such as deionized water, methanol, aqua regia, and potassium bichromate were not very effective. The intensity of the characteristic signal at 701 cm^−1^ decreased rapidly after each cleaning (Fig. S5).

Because of the stable signal intensity at 701 cm^−1^, the concentrated piranha solution (95% sulfuric acid/30% hydrogen peroxide solution (v/v = 7:3)) [8] was selected as the cleaning mixed solvent. Because of the strongly oxidizing properties of the cleaning solution, the AuNPs and other contaminants were oxidized and effectively removed from the glass vials.

**References in Text S1**

1. De Jesus MA, Giesfeldt KS, Sepaniak MJ (2004) Improving the analytical figures of merit of SERS for the analysis of model environmental pollutants. J Raman Spectrosc 35: 895-904.

2. Munro CH, Smith WE, Garner M, Clarkson J, White PC (1995) Characterization of the surface of a citrate-reduced colloid optimized for use as a substrate for surface-enhanced resonance Raman scattering. Langmuir 11: 3712-3720.

3. Laserna JJ, Torres EL, Winefordner JD (1987) Studies of sample preparation for surface-enhanced Raman spectrometry on silver hydrosols. Anal Chim Acta 200: 469-480.

4. Cook JC, Cuypers CMP, Kip BJ, Meier RJ, Koglin E (1993) Use of colloidal SERS for the trace detection of organic species: a survey based on pyridine as the probe molecule. J Raman Spectrosc 24: 609-619.

5. Schneider S, Grau H, Halbig P, Freunscht P, Nickel U (1996) Stabilization of silver colloids by various types of anions and their effect on the surface-enhanced Raman spectra of organic dyes. J Raman Spectrosc 27: 57-68.

6. Freeman RG, Bright RM, Hommer MB, Natan MJ (1999) Size selection of colloidal gold aggregates by filtration: Effect on surface-enhanced Raman scattering intensities. J. Raman Spectrosc 30: 733-738.

7. Ratna T, Brown RJC, Milton MJT (2007) Strategy to improve the reproducibility of colloidal SERS. J Raman Spectrosc 38: 1469-1479.

8. Princeton Univesity (2014) Section 10: Chemical Specific Information Piranha Solutions. Available online: http: //web.princeton.edu/sites/ehs/labsafetymanual/cheminfo/piranha.htm (accessed on 9 April 2007).
